# Structural Equation Model Analysis of HIV/AIDS Knowledge, Attitude, and Sex Education Among Freshmen in Jiangsu, China

**DOI:** 10.3389/fpubh.2022.892422

**Published:** 2022-05-18

**Authors:** Fulai Tu, Ruizhe Yang, Rui Li, Guoping Du, Yangyang Liu, Wei Li, Pingmin Wei

**Affiliations:** ^1^Key Laboratory of Environmental Medicine Engineering, Department of Epidemiology and Health Statistics, School of Public Health, Southeast University, Nanjing, China; ^2^Department of Prevention and Health Care, Children's Hospital of Nanjing Medical University, Nanjing, China; ^3^Department of General Practice, Southeast University Hospital, Nanjing, China; ^4^Department of Quality Management, Children's Hospital of Nanjing Medical University, Nanjing, China

**Keywords:** acquired immunodeficiency syndrome (AIDS), human immunodeficiency viruses (HIV), structural equation model, knowledge attitude and sex education, freshmen

## Abstract

**Background:**

The study of acquired immunodeficiency syndrome (AIDS) related knowledge, attitude, and sex education status of Jiangsu freshmen was conducted, which can provide data support directionally for the prevention work of HIV/AIDS among this population.

**Methods:**

Male students (4,006) and female students (4,279) were selected from 20 universities or colleges in the Jiangsu province. The knowledge, attitudes, and sex education of freshmen were conducted with an online questionnaire. The log-binomial regression model was used to analyze the influencing factors of HIV/AIDS knowledge. In addition, a structural equation model was used to analyze students' health needs that affect knowledge awareness and knowledge mastery.

**Results:**

The overall awareness rate of AIDS knowledge was 87.4%. The students in undergraduate colleges (OR = 2.523, 95% CI=2.223~2.864) and independent colleges (OR = 1.389, 95%CI = 1.172~1.646) were more likely to have a higher awareness compared with the students in junior colleges. In this study, 2,011 freshmen approved of premarital behavior, 4,921 freshmen insisted on using condoms when having sex, and 8,138 freshmen were willing to take HIV antibody test when they suspected they were infected. In total, 4,703 freshmen believed that sexual health education was necessary for colleges and universities, and most of them (57.2%) hoped that sex education in schools should be improved. The direct effect of sex education on knowledge awareness and attitude is 0.15 and 0.58. The mediation effect test found that the pass ability knowledge path of sex education indirectly affected sexual attitudes (0.05).

**Conclusion:**

The awareness rate of HIV/AIDS among Jiangsu freshmen has not reached the national standard. Health education has a significant positive effect on knowledge awareness and attitude; however, students' needs in terms of time, place, and degree of sex education have not been met in time. It is necessary to strengthen the HIV/AIDS health education of college students in multiple ways.

## Introduction

As one of the vastest countries that contains a quarter of the world's population, China had faced various complex challenges in its fight against HIV/AIDS. The epidemic of HIV/AIDS in China mainly experienced three stages: sporadic cases (1985–1988), endemic outbreaks (1989–1994), and expansion phase (1995 to present). China has made remarkable achievements in HIV preventions and controls during the past three decades (1). The previous studies had indicated that higher risk existed among the young- and middle-aged populations, especially for the population who are aged between 30 and 40 years (2), MSM is the highest-risk group for HIV infection in the country (3), and people of lower socioeconomic status, unemployed populations, business workers, and rural labors are usually considered as the groups which had the highest opportunity be affected by HIV/AIDS frequently (4).

Despite substantial progress in understanding and treating HIV/AIDS (5), HIV remains a major global concern (6), HIV/AIDS has become one of the main causes of death among teenagers. Over 300 children and adolescents die every day from AIDS-related causes (7). Although China's HIV/AIDS epidemic remains low-prevalence level, the number of young students who are infected with HIV still keeps rising (8). From 2010 to 2019, China has reported a total of over 140,000 cases of HIV infection among young students between 15 and 24 years (9). In the corresponding years, the proportion of the total population of infected young students had increased from 8.5% in 2010 to 21.7% in 2019 (10), which shows HIV/AIDS prevention situation among young students is severe.

Recently, increased HIV infections among college students is one of primary challenges for the overall HIV prevention strategies in China (11). As an important educational center, Jiangsu province has a total of 168 higher education institutions and almost 2.25 million students, which ranking the first of all the Chinese provinces. Spatial analysis had shown geographic variations of HIV infection among young population in China, and the cumulative number of new HIV diagnoses among young students in Jiangsu province ranks in the top three in China from 2010 to 2019 (9). Although the Jiangsu government announced an implementation plan for preventing the spread of HIV/AIDS in the province (2019–2022) (12), there is still much space for improvement in the terms of adolescent HIV/AIDS prevention and health education (13). Meanwhile, due to the lack of self-motivation for prevention and treatment among the target population, departments of education and health require to work together in policy making for HIV prevention and care among college students on campus (14).

With the rapid development of social economic culture, young students tend to open sexual concepts gradually, and sexual behaviors become pervasive. They are likely to be imitation and conformity (15). However, inadequate sexual knowledge and some risky sexual behaviors (i.e., reduced use of the condom, especially with casual partners, and sexual intercourse with unknown persons) are quite diffused among young people (15).

There is good evidence from many countries that comprehensive sex education programs lead to safer sexual behaviors. However, the implementation of sex education in China is still inadequate (16). In light of the increased prevalence of HIV/AIDS among young students, preventive education is an integral and basic part of an effective and comprehensive combination HIV prevention program. Hence, effective knowledge and education programs should be mainstreamed across universities and schools to prevent new HIV infections (17).

Empirical evidence suggests mixed reports regarding the level of HIV/AIDS knowledge, attitude, and practice among university students (18), previous studies on students' HIV/AIDS knowledge, attitudes, and behaviors among undergraduates university and college students (19–21), and few studies have conducted HIV-/AIDS-related KAP survey among the freshmen. The purpose of this study is to understand the knowledge, attitudes, and sex education of freshmen from the Jiangsu colleges and universities, we also analyzed factors affecting the overall awareness rate of knowledge about HIV/AIDS and explore the relationship between sex knowledge, attitudes, and education. To our knowledge, this is the first study in a province-wide survey of a large sample, helping to provide a theoretical basis for the further development of HIV/AIDS prevention among college and university students.

## Methods

### Ethics Approval and Consent to Participate

This study was reviewed and approved by the Human Research Ethics Committee of the Zhongda Hospital affiliated to the Southeast University, China (approval ID: 2017ZDKYSB045). All the participating subjects received written detailed information on the study, and signed consent forms for the interview and the processing of sensitive personal data, if the participants were younger than 18 years, parental informed consent was obtained. The procedure of the study was performed following the guidelines outlined in the Declaration of Helsinki.

### Participants

Freshmen who come from the class of 2020 are incorporated as the research objects. In total, 20 universities and colleges in the Jiangsu Province are randomly selected through stratified clusters (including three independent colleges, 12 undergraduate universities, and five technical colleges). Second, 10–13 classes were selected randomly from the freshman of each university. Finally, 16–18 male and female students of the Jiangsu domicile were randomly selected from each class as the research object. A total of 8,580 students were selected and 8,285 valid questionnaires were collected. The effective rate is 96.6%.

### Data Collection

A self-made structural questionnaire was applied, and the content of the survey include: (1) basic demographic characteristics, including age, gender, school, region, parental education, family income, living style, etc.; (2) AIDS-related knowledge, which was designed refer to 8 basic AIDS prevention knowledge of young students in China. Awareness is judged by answering 6 or more questions correctly; (3) AIDS-related sexual attitudes, including sexual behaviors and sexual education; (4) current status of AIDS health education.

### Quality Control

Producing standardized electronic questionnaires, and conducting online surveys through the health data management system for freshmen in the universities of Jiangsu Province. Professionals from the health departments of various universities served as quality controllers. They were uniformly trained before the survey, the training that required them to clarify survey requirements, be familiar with the process, and stress the standard of an effective questionnaire which should include complete demographic data, no outliers, and less than four missing items of principal part. During the survey, the respondents were gathered by counselors in a computer lab, and filled out an online questionnaire anonymously.

### Statistical Analysis

The SPSS 22.0 statistical software (SPSS Inc. Chicago IL, USA, 2013) was used for data analysis, chi-square test was used for rate comparison. In cross-sectional studies, when the incidence of outcome is high (e.g., >10%), the correlation strength between outcome and factors may be overestimated if logistic regression model is used to calculate odds ratio (odds ratio, OR). In this case, log-binomial regression model should be used to calculate the prevalence ratio (prevalence ratio, PR) and carry out multivariate analysis with the test level of 0.05. The structural equation model was constructed by the SPSS AMOS 25.0 software.

## Results

### Basic Demographic Information

Among 8,285 survey subjects, 4,006 were men (48.4%). The age distribution ranged from 15 to 25 years old, more than 65% of the participants were 18 years old or younger. There are 1,850 (22.3%) from the technical colleges, 5,130 (61.9%) from undergraduate universities, and 1,305 (15.8%) from independent colleges. According to the geographical location of the cities under the jurisdiction of the Jiangsu Province, this study divides the Jiangsu Province into Southern Jiangsu (Nanjing, Zhenjiang, Changzhou, Wuxi, Suzhou), Central Jiangsu (Nantong, Taizhou, Yangzhou), and Northern Jiangsu (Yancheng, Huai'an, Lianyungang, Xuzhou and Suqian), among all the participants, 3,102 (37.4%) are from South Jiangsu, 1,931 (23.3%) are from Central Jiangsu, and 3,252 (39.3%) are from North Jiangsu.

### Awareness of Sexual Knowledge Among Freshman Students

In the total eight questions, “After high-risk behaviors (sharing needles, drug use, unsafe sex, etc.), should actively seek HIV testing and counseling” has the highest awareness rate, which is 98.0%; “At present, whether the main mode of transmission of AIDS among young students in our country is male homosexual behavior, followed by heterosexual behavior” has the lowest awareness rate, which is 60.9%. The total awareness rate of AIDS knowledge among 8,285 participants is 87.4%. There are statistically significant differences about the awareness rate of AIDS knowledge among different genders, schools, regions, whether they are or not only child, parent education level, family monthly income, and residence style (*P* < 0.05), see Table 1. By adopting AIDS knowledge awareness rate (know = 1, do not know = 0) as a dependent variable, and the aforementioned statistically significant (*P* < 0.05) factors as independent variables, a log-binomial regression model was constructed. The results show that the overall awareness rate of AIDS among freshmen of undergraduate universities is higher than that of junior colleges (OR = 2.523, 95% CI=2.223~2.864), and independent colleges are higher than junior colleges as well (OR = 1.389, 95%CI = 1.172 ~1.646); see Table 2.

**Table 1:** Single factor analysis of the overall awareness rate of AIDS knowledge among freshmen with different demographic characteristics.

**Demographic indicators**	**Number of awareness *n*(%)**	**χ^2^ Value**	***P*-Value**
Gender		4.291	0.038
Male(*n =* 4006)	3532(88.2)		
Female(*n =* 4279)	3708(86.7)		
School		265.579	<0.001
Junior college(*n =* 1850)	1416(76.5)		
Undergraduate college(*n =* 5130)	4679(91.2)		
Independent college(*n =* 1305)	1145(87.7)		
Region		7.315	0.026
Southern Jiangsu(*n =* 3102)	2750(88.7)		
Central Jiangsu(*n =* 1931)	1669(86.4)		
Northern Jiangsu(*n =* 3252)	2921(87.4)		
Only child		24.593	<0.001
Yes(*n =* 5005)	4447(88.9)		
No(*n =* 3280)	2793(85.2)		
Father's education level		38.796	<0.001
Junior high and below(*n =* 3543)	3013(85.0)		
High school(*n =* 2362)	2078(88.0)		
College/undergraduate or above(*n =* 2380)	2149(90.3)		
Mother's education level		53.554	<0.001
Junior high and below(*n =* 4176)	3554(85.1)		
High school(*n =* 2331)	2082(89.3)		
College/University and above(*n =* 1778)	1604(90.2)		
Family monthly income		27.268	<0.001
<1000(*n =* 205)	175(85.4)		
1000~3000(*n =* 871)	740(85.0)		
≥3000(*n =* 7209)	1681(85.0)		
Living style		21.336	0.001
Live with parents(*n =* 4308)	3828(88.9)		
Others(*n =* 3977)	144(82.3)		

**Table 2:** Log-binomial regression analysis of total awareness rate of AIDS knowledge among freshmen.

**Independent variable**	**β Value**	**Standard value**	**95%*Wald* Confidence interval**	**Hypothetical test**			**Exp(β)**	**95%Exp(β)*Wald*** **Confidence interval**
				***Wald* χ^2^ Value**	**df**	**P Value**		
School								
Junior college	Ref	Ref	Ref	Ref	Ref	Ref	Ref	Ref
Undergraduate college	0.925	0.065	(0.799~1.052)	205.198	1	<0.001	2.523	(2.223~2.864)
Independent college	0.329	0.087	(0.159~0.498)	14.395	1	<0.001	1.389	(1.172~1.646)

### Freshman Sexual Attitudes

#### Attitudes About Sexual Behaviors

Among 8,285 survey respondents, 2,011 (24.2%) students are in favor of premarital sex, and 4,921 (59.4%) are willing to insist on using condoms during sexual behaviors, 8,138 (98.2%) students are willing to be tested for HIV antibodies when they suspect they are infected. The aforementioned sexual attitudes have statistically significant differences in gender, school, and family monthly income/person (all *P* < 0.05). According to chi-square segmentation pairwise comparison, students who come from the undergraduates' college are more likely in favor of premarital sex than junior college students, students who come from the Southern Jiangsu areas are more likely in favor of premarital sex than students come from the Northern Jiangsu areas. Compared with junior college students, undergraduate college students are more willing to insist on using condoms and taking HIV antibody test if they suspect they had been infected. Students who come from the Southern Jiangsu areas are more willing to insist on using condoms and taking HIV antibody tests if they suspect they had been infected (all *P*-values <0.05). The results of the rest pairwise comparison is shown in Table 3.

**Table 3:** Different demographic characteristics of freshmen attitudes about sexual behaviors.

**Items**	**Accept premarital sex**			**Insist on using condom during sexual behaviors**			**Willing to do HIV antibody test**		
	**Constituent ratio *n*(%)**	**χ^2^ value**	***P* value**	**Constituent ratio *n*(%)**	**χ^2^ value**	***P*-value**	**Constituent ratio *n*(%)**	**χ^2^ value**	***P*-value**
**Gender**		499.009	<0.001		491.891	<0.001		30.058	<0.001
Male	1408(35.1)			1884(47.0)			3902(97.4)		
Female	603(14.1)			3037(71.0)			4236(99.0)		
**School**		6.900	0.032		63.001	<0.001		13.588	0.001
Junior college	411(22.2)			971(52.5)			1802(97.4)		
Undergraduate college	1292(25.2)^a^			5060(98.6)^a^			3212(62.6)^a^		
Independed college	308(23.6)			738(56.6)			1276(97.8)		
**Region**		12.674	0.002		48.592	<0.001		1.724	0.422
Northern Jiangsu	738(22.7)			1809(55.6)			3202(98.5)		
Central Jiangsu	454(23.5)			1124(58.2)			1894(98.1)		
Southern Jiangsu	819(26.4)^c^			3042(98.1)			1988(64.1)^bc^		
**Family monthly income**		6.642	0.036		10.418	0.005		11.662	0.003
<1000	48(23.4)			110(53.7)			195(95.1)		
1000~3000	181(20.8)			481(55.2)			857(98.4)^*d*^		
≥3000	1782(24.7)^f^			4330(60.1)^f^			7086(98.3)^e^		
**Living style**		6.145	0.013		33.470	<0.001		0.328	0.567
Living with parents	1094(25.4)			2688(62.4)			4235(98.3)		
Others	917(23.1)			2233(56.1)			3903(98.1)		

*By pairwise comparison of chi-square method, “a” means P < 0.01 when junior college compares to undergraduate college; “b” means P < 0.01 by comparing southern Jiangsu areas to central Jiangsu areas, “c” means P < 0.01 by comparing southern Jiangsu to northern Jiangsu; “d” means P < 0.01 by comparing family monthly income of 1000—3000 RMB with income of 1000 RMB or less, “e” means family monthly income of 1000 yuan or less compared with 3000 yuan or more, P < 0.01, “f” means P < 0.01 by comparing family monthly income of 1000—3000 RMB with income of 3000 RMB or more*.

#### Attitudes of Sexual Education Development

Among the 8,285 survey participants, 3,441 (41.5%) freshmen believe that the reasonable time to conduct sexual health education is during junior high school, 4,703 (56.8%) freshmen believe that colleges and universities must carry out sexual health education, and most of them (57.2%) hope that the intensity of sex education in schools should be improved. In addition, 2,238 (27.0%) freshmen believe that school should conduct sex education in multiple ways by offering special, elective courses and additional lectures, increasing activity promotions, distributing brochures or other methods; 1,391 (16.8%) freshmen believe that in addition to publicizing AIDS prevention knowledge, it is necessary to comprehensively add sexual physiology and psychology knowledge, sexual ethics and sexual crimes knowledge, heterosexual and contraceptive knowledge, and how to avoid being sexually assaulted relevant knowledge.

#### Sex Education Status of Freshmen

**Figure 1:**
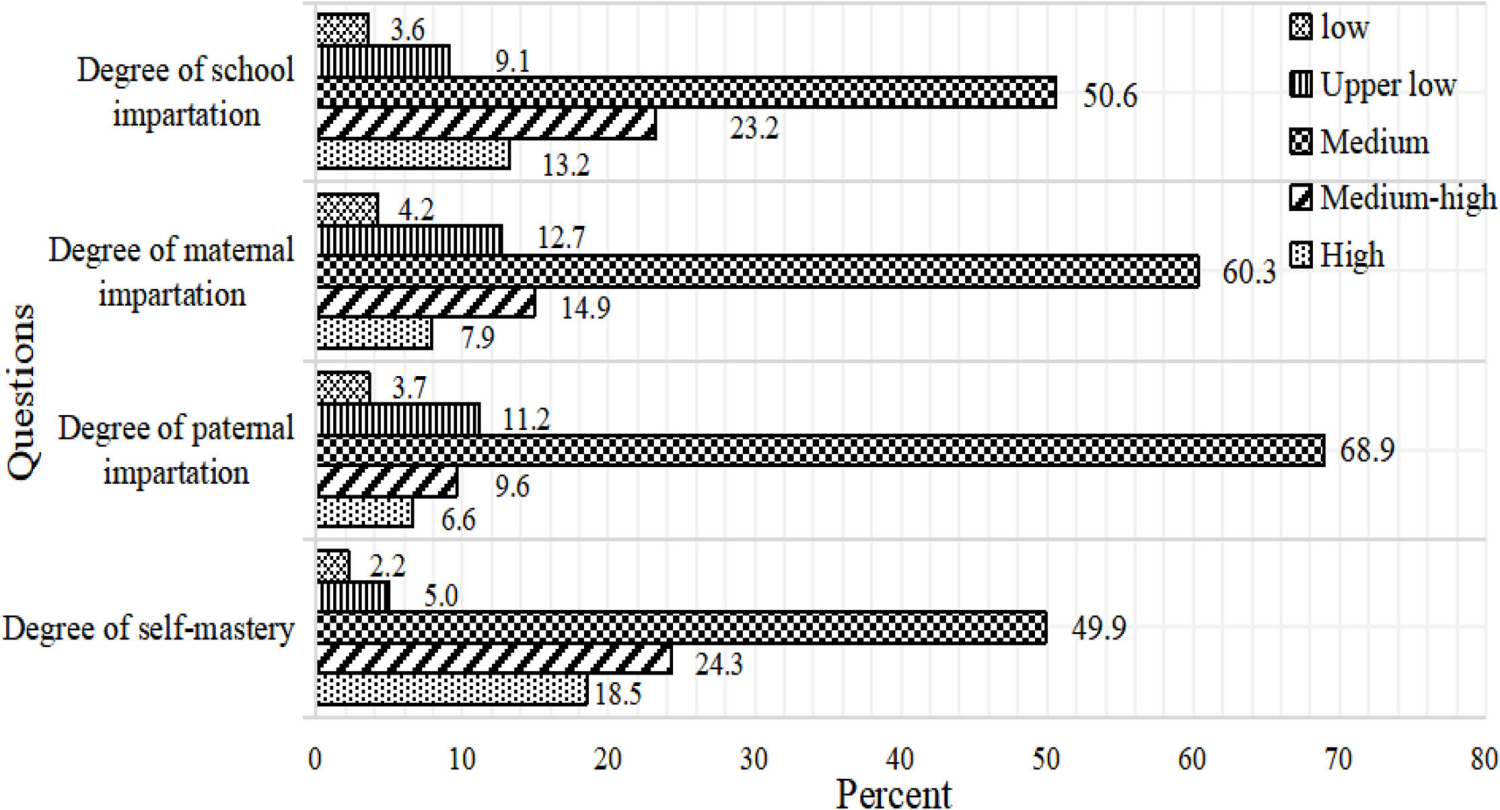
Sex education status of freshmen.

Among 8,285 survey participants, 3,092 (37.3%), 2,881 (34.8%), and 2,301 (27.8%) freshmen indicated that they just received general sexual education from school and parents. Nearly half (49.9%) of the freshmen believe that they have only received sexual education at the general level; see Figure 1.

#### The Relationship Between Sex Knowledge, Attitudes, and Education

Taking sex knowledge, attitude, and education as latent variables, through exploratory factor analysis, the observation variables in Table 4 are incorporated to construct a structural equation model. The Cronbach's α coefficient of this model is 0.696, the KMO value is 0.708, the approximate chi-square value of the Bartlett sphericity test is 17,649.686, the df is 78, and the *P*-value is < 0.001.

**Table 4:** Latent variable and observational variable.

**Latent variable**	**Observational Variable**
Sexual education	Q1 What do you think the level of sexual education that your school taught you
	Q2 What do you think about the level of sex education you have received
	Q3 What do you think the level of sexual education that your father taught you
	Q4 What do you think the level of sexual education that your mother taught you
Sexual knowledge	Q5 Can insist on using condoms correctly reduce the risk of infect and spread HIV?
	Q6 Does the use of new-type drugs increase the risk of AIDS?
	Q7 Should we actively look for HIV testing and counseling after high-risk behaviors?
	Q8 Are the rights of HIV-infected patients in marriage, employment and school enrollment protected by the laws of our country?
Sexual attitude	Q9 Is it necessary to carry out sexual and reproductive health education in high school?
	Q10 Is it necessary for colleges and universities to carry out sexual and reproductive health education?
	Q11 Do you think the sexual and reproductive health education in your high school needs to be further improved?
	Q12 Are you willing to use condoms when you have sex with your partner?
	Q13 If you suspect that have been infected HIV, will you go for an antibody test immediately?

**Figure 2:**
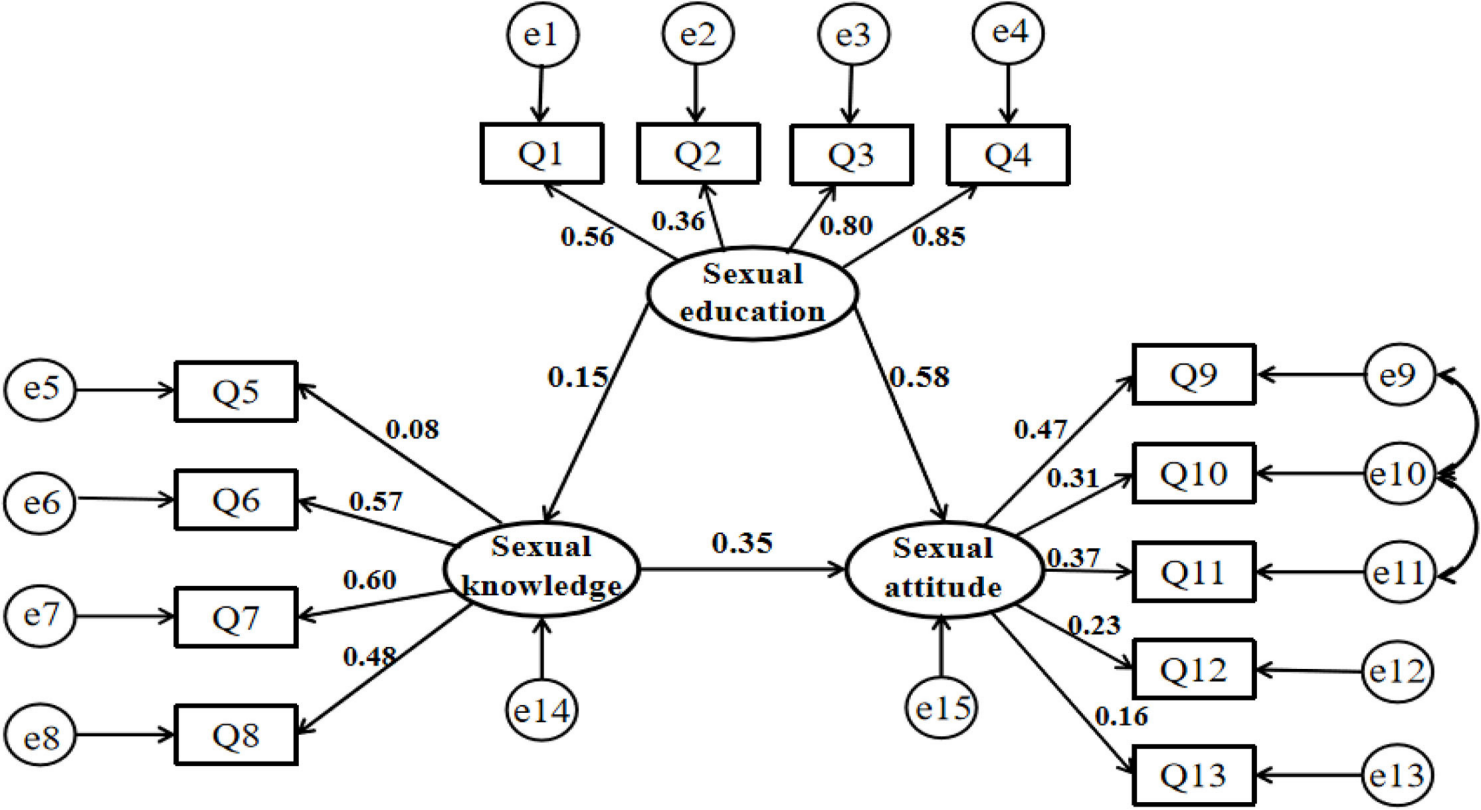
Diagram of structural equation model.

According to the value of the revised index MI, the model has proceeded with the second correction, and the paths are increased one by one: e9⇔e10, e10⇔e11. The Root Mean Square Error of Approximation (RMSEA) of the corrected model is 0.059 (<0.080), Goodness-of-Fit Index (GFI), Adjusted Goodness-of-Fit Index (AGFI), Incremental Fit Index (IFI), Comparative Fit Index (CFI) is 0.968, 0.951, 0.901, and 0.901 (both>0.900), which matches the requirements of model testing and goodness-of-fit. It shows that the overall fitting effect of the model is acceptable. Path analysis of the model shows that sex education has a significant positive effect on sex knowledge and attitudes, of which the direct effect value is 0.15, and 0.58, respectively. By using the Bootstrap method to test the intermediary effect, it is found that sexual education can indirectly affect sexual attitudes through the sexual knowledge path, and the indirect effect value is 0.05, 95% CI = 0.04~0.07, *P* < 0.001, which could prove intermediary effect is significant. See Table 5 and Figure 2.

**Table 5:** Model results of each path.

**Paths**	**Coefficient**	**Standardized coefficients**	**S.E**.	**C.R**.	***P*-value**
Sexual knowledge < - Sexual education	0.07	0.15	0.01	9.10	<0.001
Sexual attitude < - Sexual education	0.35	0.58	0.02	23.26	<0.001
Sexual attitude < - Sexual knowledge	0.46	0.35	0.04	13.34	<0.001
Q1 < – Sexual education	1.00	0.56	–	–	–
Q2 < – Sexual education	0.47	0.36	0.02	27.55	<0.001
Q3 < – Sexual education	1.48	0.80	0.03	48.07	–
Q4 < – Sexual education	1.54	0.85	0.03	48.01	<0.001
Q8 < – Sexual knowledge	1.00	0.48	–	–	–
Q5 < – Sexual knowledge	0.05	0.08	0.01	5.76	<0.001
Q6 < – Sexual knowledge	0.91	0.57	0.04	24.61	<0.001
Q7 < – Sexual knowledge	0.50	0.60	0.02	24.33	<0.001
Q9 < – Sexual attitude	1.00	0.47	–	–	–
Q10 < – Sexual attitude	0.48	0.31	0.03	16.43	<0.001
Q11 < – Sexual attitude	0.60	0.37	0.03	48.07	<0.001
Q12 < – Sexual attitude	0.27	0.23	0.02	13.82	<0.001
Q13 < – Sexual attitude	0.05	0.16	0.01	10.15	<0.001

## Discussion

This study found that the awareness rate of AIDS knowledge among freshmen of Jiangsu who came from the class of 2020 is 87.4%, which is lower than that of Shanghai (92.1%) (22), Wenzhou (96.38%) (23), Nanning (96.78%) (24), and other regions (25). The result shows the awareness rate of prevention and control knowledge for young- and middle-aged students has not reached the requirement yet, which is 90% or more than “China's 13th Five-Year Plan for Containment and Prevention of AIDS” indicates. Our study revealed that significant number of freshmen have poor knowledge regarding HIV. This suggests that the current HIV/AIDS-related health education in middle school, high school, and the entrance section of university need to be further strengthened. This can help boost the level of HIV/AIDS knowledge as well as increase HIV testing and skills for HIV prevention (26).

“At present, the prevalence of AIDS among young students in our country is showing a rapid growth trend. The main mode of transmission is homosexuality, followed by heterosexuality” has the lowest awareness rate among eight topics, only 60.9%, and that is consistent with some published reports in China (27, 28). It reminds that students' understanding of the current epidemic trend and transmission routes of AIDS is still on the surface, and it is necessary to focus on adjusting and improving the direction of AIDS knowledge publicity by increasing the strength and depth of publicity and education for weak parts. As game-based learning and gamification were used to improve the sexual health education of adolescent students (29). The results of log-binomial regression analysis which affects the awareness rate of AIDS suggest undergraduate college students are better than junior colleges and independent colleges. This reflects the factors such as students' cultural literacy, sexual education investment, and attention in school have a positive significance in prevention of AIDS. In this study, the awareness rate of AIDS did not differ by gender, schools, whether they are or not only child, parent education level, family monthly income, and residence style, this finding conflicts with some earlier researches (30, 31). Future studies may wish to assess a wider range of influence factors to better understand which affects the awareness rate of AIDS.

Previous studies have found positive correlations between HIV attitude and behavior (32). However, in the current study, the participants in this survey are university and college freshmen who have a relatively conservative attitude toward premarital sex, the percentage of respondents who are willing to insist on using condoms during sex is relatively low (59.1%). People with a longer duration of premarital sex had higher odds of HIV and other STIs and were more likely to report multiple sexual partners (33). It prompts the urgent need to increase the awareness of self-protection among freshmen by popularizing AIDS-related protective measures.

Compared with women, men are more likely to have more permissive perceptions about sex (34). Our previous study demonstrated that only 62.5 and 66.3% of HIV-positive college male students who used condoms consistently during sexual intercourse with regular and casual partners, respectively (35). Meanwhile, the willingness of HIV antibody testing among male students if they suspect that they have been infected with HIV was lower than women (36). These findings are supported by the previous studies (13, 37), this may be because of the openness of men's sexual viewpoints. HIV education services in the Chinese colleges and universities should be designed and provided comprehensively taking into consideration the needs of male undergraduates (38).

Although undergraduate students are more likely to have premarital sex compared with the junior colleges, students who come from the Southern Jiangsu are more likely to have premarital sex than middle and Northern Jiangsu's students as well, all of them are more willing to insist on using condoms and taking HIV antibody testing when having sex. This finding shows that there are big differences in the effects of sexual education between schools and regions. The imbalances in the cultivation and guidance of ideas, education services, information resources, and economic development among schools or regions should be fully considered, and should appropriately increase investment in educational resources of junior colleges and Northern Jiangsu regions.

Some researchers have pointed out that to reduce the risk of infection among young students, the most important thing is to promote comprehensive sexual education (39). The structural equation model shows that sexual education can directly have a significant positive effect on sexual knowledge (0.15) and sexual attitudes (0.53). It can also indirectly influence sexual attitudes (0.05) through the sexual knowledge pathway. However, the survey shows that more than half of the students believe that the main responsible body for reproductive and sexual health education are parents and schools, and the level of sexual education obtained from parents and schools is just the modest and nearly half of students believe that their level of sexual education is far from enough. It shows the students' needs about the duration, place, and degree of sexual education have not been satisfied in time. This not only reveals the importance and urgency of carrying out sexual education, but also reminds us when we carry out AIDS health education, we should focus on combining the function of individuals, families, and schools to make their respective advantages to jointly promote prevention work. It recommends that the duration of sexual education should be moved forward, not just slogans. Colleges and universities can organize lectures for the parent by offering sexual health education for parents first, and parents should be guided on how to conduct sexual health education for their children in the family. Meanwhile, increasing channels for students to understand HIV/AIDS-related knowledge, such as opening professional lecture and elective courses, making full use of new media network platforms (40), conducting knowledge competitions, expert interviews, and other activities to meet the needs of students in all aspects of sexual education, grasp relevant knowledge in time and ultimately guide young adults to develop positive sexual attitudes and healthy sexual behaviors.

Proceeding a survey about the status of sexual education for universities and colleges, freshmen can objectively understand the level of sexual education that high school graduates received and the situation of sexual education at the middle school stage. According to that, correct direction for effective sexual education at the university stage can be provided, and it plays a forward role in epidemic prevention and control. Many pieces of national policy of school-based health education programs on HIV/AIDS have been advocated for a long time in China (30). HIV/AIDS education was found effective in promoting positive behavior change related to HIV/AIDS prevention (41), while policy's ability to structure implementation was at a moderate level (42). In summary, the work of HIV/AIDS health education for university and college students in the Jiangsu Province needs to be strengthened. It is necessary to develop school-based culturally relevant comprehensive sexual health education programs according to various characteristics of freshmen (43). By focusing on strengthening popularization of HIV/AIDS epidemic situation and self-protection measures, establishing a personal–family–school–social relationship combining prevention and control mechanisms to effectively train university and college students' ability in the HIV/AIDS prevention field.

## Conclusion

The awareness rate of HIV/AIDS among the Jiangsu freshmen has not reached the national standard. Health education has a significant positive effect on knowledge awareness and attitude; however, the students' needs in terms of time, place, and degree of sex education have not been met in time. It is necessary to develop school-based culturally relevant comprehensive sexual health education programs according to various characteristics of freshmen.

### Strengths and Limitations of This Study

The number of young students infected with HIV is still rising in China, and HIV/AIDS has become one of the leading causes of death among young people. Therefore, we chose the student population as the research object. From a regional perspective, the Jiangsu province, with its rapid economic development, convenient information exchange, frequent population mobility, and active employment in other places has created conditions for the spread of HIV. It is also an important hot spot for young people infected with HIV. The large sample size across the various cities presents us with an opportunity to determine disparities in prevalence within a province. From the perspective of methodology, the log-binomial method is reasonably used in our study, which is different from the logistic regression method used in most studies in terms of multi-impact factor analysis. For the first time, we use the structural equation model to better understand the students' health needs that affect knowledge awareness and knowledge mastery. Finally, in the terms of research significance, our study focuses on the relationship between sex education and knowledge and attitude, which is different from the common research purpose of knowledge, belief, and behavior. There are several limitations to our study. At first, the information was collected using a self-administered questionnaire. Second, the honesty and the seriousness of the respondents to the questions are difficult to access and validate. Third, we did not investigate the education level of students in high school, but we believe that the region of students can reflect the education level of their high school, Southern Jiangsu, central Jiangsu, and northern Jiangsu represents three levels of education high, medium, and low, respectively. Lastly, we did not investigate the regional origin (urban and rural), sexual orientation of the respondents, the bias may occur in the results.

## Data Availability Statement

The raw data supporting the conclusions of this article will be made available by the authors, without undue reservation.

## Ethics Statement

The studies involving human participants were reviewed and approved by this study was reviewed and approved by the Human Research Ethics Committee of the Zhongda hospital affiliated Southeast University, China (approval ID: 2017ZDKYSB045). Written informed consent to participate in this study was provided by the participants' legal guardian/next of kin.

## Author Contributions

FT: methodology, software, formal analysis, and writing. RY: conceptualization, project administration, and writing. RL: data curation. GD: writing. YL: methodology. WL and PW: conceptualization, supervision, and writing. All authors contributed to the article and approved the submitted version.

## Funding

This work was supported by Jiangsu Provincial Health Development Center Open Project in 2021 (JSHD2021018) and the Key Research Project of Jiangsu Province's 14th Five-Year Plan Higher Education Scientific Research Plan (ZDDY12).

## Conflict of Interest

The authors declare that the research was conducted in the absence of any commercial or financial relationships that could be construed as a potential conflict of interest.

## Publisher's Note

All claims expressed in this article are solely those of the authors and do not necessarily represent those of their affiliated organizations, or those of the publisher, the editors and the reviewers. Any product that may be evaluated in this article, or claim that may be made by its manufacturer, is not guaranteed or endorsed by the publisher.
